# Biomarker Evaluation in Young Onset Dementia from Diverse Populations

**DOI:** 10.1002/alz.093025

**Published:** 2025-01-09

**Authors:** Eden V. Barragan, Hilary W. Heuer, Rachel L. Nosheny, Monica R. Camacho, Krista Navarra, Paul S. S. Aisen, Annalise Rahman‐Filipiak, J. Scott Roberts, Adeyinka Ajayi, Desiree Byrd, Monica Rivera Mindt, Gil D. Rabinovici, Adam L. Boxer

**Affiliations:** ^1^ University of California, San Francisco, San Francisco, CA USA; ^2^ VA Advanced Imaging Research Center, San Francisco Veterans Affairs Medical Center, San Francisco, CA USA; ^3^ Northern California Institute for Research and Education (NCIRE), San Francisco, CA USA; ^4^ Alzheimer's Therapeutic Research Institute, University of Southern California, San Diego, CA USA; ^5^ Michigan Alzheimer's Disease Research Center ‐ University of Michigan, Ann Arbor, MI USA; ^6^ University of Michigan School of Public Health, Ann Arbor, MI USA; ^7^ Icahn School of Medicine at Mount Sinai, New York, NY USA; ^8^ CUNY, Queens College, Queens, NY USA; ^9^ Fordham University, New York, NY USA; ^10^ Memory and Aging Center, Weill Institute for Neurosciences, University of California, San Francisco, San Francisco, CA USA; ^11^ Memory and Aging Center, UCSF Weill Institute for Neurosciences, San Francisco, CA USA

## Abstract

**Background:**

Early onset dementia (EOD) affects people at the peak of their personal and professional responsibilities and economic productivity. Alzheimer’s disease (AD) and Frontotemporal Dementia (FTD) are the most common EOD etiologies in Non‐Latinx White adults (NLW). Black and Latinx older adults bear a disproportionate burden of dementia compared to NLW, likely due to vulnerabilities that confer increased risk, such as cardiovascular factors, socioeconomic stressors, and structural racism. Little is known, however, about the prevalence and causes of EOD in Black, Latinx, and other diverse populations (DP) because most EOD studies have primarily recruited NLW individuals. Many barriers impede research participation in DP, but community‐engaged research (CER) strategies to collect diagnostic data, like blood‐based biomarkers and digital assessments, may increase access and convenience of research participation in DP.

**Method:**

The BEYONDD study (www.beyonddproject.org), a new NIH‐funded study, is recruiting 200 DP individuals to evaluate the use of remote assessments and the impact of returning diagnostic information to participants as a more accessible research framework to understand the etiology of EOD in DP. Participants are recruited using CER strategies and screened via online platform for eligibility (age 40‐64, with concerns about cognitive or behavioral function). Participants complete the following procedures remotely and receive gift cards after completion: online questionnaires, self‐administered tablet‐based cognitive testing (Brain Health Assessment), and an in‐home blood draw for standard labs and plasma biomarkers of AD and neurodegeneration (Aβ and P‐tau217 ratios, NfL). Participants can learn their results remotely or in person. Participants are invited for more comprehensive onsite evaluation at one of 10 BEYONDD clinical centers, followed by appropriate referral to other NIH‐funded research programs.

**Result:**

The study has recruited its first participants. Early participant feedback suggests that remote, accessible procedures may increase DP engagement and participation in EOD research. We expect substantial enrollment by July and will report initial results and plans for refinement of study procedures.

**Conclusion:**

The importance of surmounting historic barriers to DP participation in EOD research is clear. We plan to build on the experience of this project to improve DP enrollment into ongoing and future observational research and clinical trials.

